# Does Ramadan fasting influence time-motion metrics and psychophysiological responses in soccer players during small-sided games performed in fed and fasted states?

**DOI:** 10.5114/biolsport.2026.156231

**Published:** 2025-12-16

**Authors:** Mohamed Kerkeni, Karim Chamari, Manel Kerkeni, Omar Boukhris, Achraf Ammar, David B. Pyne, Waqar Husain, Hamdi Chtourou, Abdul Rashid Aziz, Haitham Jahrami, Khaled Trabelsi

**Affiliations:** 1 High Institute of Sport and Physical Education, University of Sfax, Sfax, Tunisia; 2 Research laboratory, Education, Motricity, Sport and Health (EM2S), LR15JS01, High Institute of Sport and Physical Education, University of Sfax, Tunisia; 3 Naufar Center, Doha, Qatar; 4 Sport, Performance, and Nutrition Research Group, School of Allied Health, Human Services and Sport, La Trobe University, Melbourne, Australia; 5 SIESTA Research Group, School of Allied Health, Human Services and Sport, La Trobe University, Melbourne, Australia; 6 Department of Training and Movement Science, Institute of Sport Science, Johannes Gutenberg-University Mainz, Mainz, Germany; 7 Research Laboratory, Molecular Bases of Human Pathology, LR19ES13, Sfax, Tunisia; 8 Faculty of Health, Research Institute for Sport and Exercise, University of Canberra, Kirinari Street, Bruce, Canberra, ACT 2617, Australia; 9 Department of Humanities, COMSATS University Islamabad, Islamabad Campus, Park Road, Islamabad, Pakistan; 10 Physical Activity, Sport, and Health, National Observatory of Sport, Tunis, Tunisia; 11 Sport Science and Sports Medicine, Singapore Sport Institute, Sport Singapore, Singapore; 12 Department of Psychiatry, Ministry of Health, Manama, Bahrain; 13 Department of Psychiatry, College of Medicine and Medical Sciences, Arabian Gulf University, Manama, Bahrain; 14 Department of Movement Sciences and Sports Training, School of Sport Science, The University of Jordan, Amman-Jordan

**Keywords:** Actigraphy, Cardiovascular responses, Fatigue, Football, Global Positioning System, Intermittent fasting

## Abstract

We explored how Ramadan fasting (RF) influences GPS-derived time-motion metrics and psychophysiological responses in soccer players during small-sided games (SSG). Twelve semi-professional male players (mean age 21.1 ± 0.7 y; estimated ${\dot{\text{V}}\text{O}}_{2\max}$ 54.5 ± 2.2 mL/min/kg) participated in four experimental SSG sessions in a within-subject, counterbalanced design. These sessions were scheduled at 15:00 and 21:00 before Ramadan, in a fed state (i.e., BR15_fed_ and BR21_fed_), and during the fourth week of Ramadan at 15:00 in a fasted state (DR15_fasted_) and at 21:00 in a fed state (DR21_fed_). Sleep patterns, dietary intake, and insomnia symptoms were evaluated during the week preceding and final week-of-Ramadan. Participants’ Hooper index (wellness) and daytime sleepiness and mood measures were assessed before each session. Time-motion metrics using GPS and exercise heart rate (HR) were assessed during all sessions, and ratings of perceived exertion (RPE) were collected after each session. Except for a higher number of decelerations in DR21_fed_ session (*p* < 0.001), RF had no significant effect on other time-motion metrics. Absolute and relative exercise HR were higher at BR15_fed_ than at BR21_fed_ and at DR15_fasted_. RPE, Hooper index scores, and perceptual stress and muscle soreness components were higher at DR15_fasted_ compared to BR15_fed_. Additionally, RF was associated with decreased sleep duration (by ~45 minutes) and increased insomnia symptoms and daytime sleepiness, while mood states and dietary intake were unchanged. RF induced physiological and perceptual changes in response to exercise, particularly in the fasted state. However, SSG performance metrics remained stable, suggesting fasted athletes can maintain short-duration SSG performance regardless of fasting status.

## INTRODUCTION

Healthy pubescent Muslims observe fasting during Ramadan, which is the ninth month of the Islamic calendar [1]. During this month, Muslims fast daily for 29–30 days every year, abstaining from eating, drinking, smoking, and other activities, such as sexual intimacy, from dawn to sunset [2, 3]. This fasting schedule introduces unique challenges for athletes, as it alters regular eating, drinking, and exercise or training recovery patterns, which are essential for maintaining physical performance and well-being [4]. Physical exertion during training or competing while fasting can intensify these challenges, leading to additional psychophysiological stress [1].

Numerous studies have been conducted to investigate the effects of Ramadan fasting (RF) on athletic performance and identify potential factors contributing to any observed performance decline [5–7]. Systematic reviews with or without meta-analyses indicate that highintensity performance metrics, such as mean and peak power outputs during the 30-sec all-out anaerobic Wingate cycle test are negatively affected when measured in the afternoon [8], while endurance performance also declines in soccer players, likely due to progressive hypohydration throughout the fast [3, 9].

Sleep disturbance has also been identified as a critical factor contributing to RF-related performance decline [8, 10]. This issue of poor quality and/or quantity of sleep, frequently reported during RF research studies, has been documented in systematic reviews and meta-analyses [10–12]. However, the lack of objective sleep assessments (e.g., polysomnography or actigraphy) in most of these studies limits the validity and reliability of these analyses, indicating the need for closer investigations [12]. Additionally, insomnia symptoms and daytime sleepiness tend to increase during the observance of RF, compounding performance challenges [13]. Psychological factors, including increased fatigue and decreased vigor assessed using a mood state questionnaire, have been reported during RF [2] and could contribute to decreases in physical performance. Indeed, exercise perceptual responses, such as assessed by the rate of perceived exertion (RPE), could further impact exercise performance during RF. The RPE reflects an athlete’s subjective perception of effort and is influenced by physiological, psychological, and environmental factors [14, 15]. Disruptions in energy intake, hydration, and recovery processes during RF increase the RPE, making physical tasks more challenging [16]. This mismatch between actual and perceived effort could explain the observed decline in performance, particularly during high-intensity or prolonged activities [15].

Cardiovascular responses, particularly exercise heart rate (HR), serve as a key indicator of athletic performance and training adaptation [17]. However, studies report inconsistent submaximal HR responses during RF [7, 18], likely related to interindividual variability in fitness levels, fasting duration, environmental conditions, hydration status, and/or physiological adaptation. Additional confounding factors such as fluctuating training loads, variable nutritional intake, and sleep quality may further impact exercise HR patterns. Resolving these discrepancies through a rigorous investigation of cardiovascular dynamics could enhance training and recovery strategies for athletes who concurrently engage in training and observe RF.

Notably, most studies examining the impact of RF on exercise performance rely on controlled and/or laboratory-based exercise tests, which fail to replicate the complexity of real-world competition or training scenarios [19]. Global positioning system (GPS) technology has previously been used to assess time-motion characteristics during a 90-minute soccer match in the Ramadan-fasted state, revealing a negative effect of RF on the physical activity profiles of fasted sub-elite soccer players [19]. However, this study lacked detailed of the players’ dietary intake and exercise HR data, and relied solely on questionnaires to determine players’ sleep profiles, factors critical for understanding players’ performance and recovery [20]. These limitations highlight the need for further investigations using more comprehensive and ecologically valid approaches that incorporate physiological, nutritional, and sleep-related parameters.

Small-sided soccer games (SSG) offer a practical and ecologically valid approach for evaluating high-intensity intermittent exercise performance [21]. Indeed, in soccer, the SSG is a widely recognized training tool because of its ability to integrate physical, technical, and tactical development, aligning with the demands of modern matches [22]. SSG is highly adaptable, with intensity influenced by pitch size, rule modifications, player numbers, and age [23–26]. SSG also provides a multidimensional assessment of players’ performance, simulating the physical, cognitive, and tactical challenges of real-world match conditions [27]. To the best of the authors’ knowledge, no previous studies have investigated the effects of observing RF on SSG performance, particularly when comparing exercising in the fed and fasted states during the Ramadan month. Training in a fed state during Ramadan month (i.e., after breaking the day’s fast) is considered optimal, as it ensures energy availability to support subsequent performance and recovery [28]; however, logistical constraints often require athletes to train in a fasted state during the daytime [3]. Understanding SSG performance and psychophysiological differences between fasted and fed states can help optimize training strategies aligned with athletes’ capacities during periods of daytime fasting in the Ramadan month.

We investigated the effect of RF on GPS-derived time-motion metrics and psychophysiological responses during SSG in soccer players. We further examined the differences in outcomes between sessions conducted in the fed (evening) and fasted (daytime) states during RF. We expected that RF would negatively affect both timemotion metrics and psychophysiological responses, with more pronounced effects observed in the fasted state (i.e., daytime exercise) compared to the fed state (evening exercise).

## MATERIALS AND METHODS

### Participants

Twelve semi-professional male soccer players (mean ± SD; age: 21.1 ± 0.7 y; stature: 176 ± 8 cm; body mass: 66.4 ± 5.5 kg; fat percentage: 6.9 ± 3.1%; muscle mass: 58.7 ± 2.6 kg; estimated ${\dot{\text{V}}\text{O}}_{2\max}$: 54.5 ± 2.2 mL/min/kg) voluntarily participated. G∗power software [29] was used to calculate the sample size a priori, following Beck’s [30] procedures. With a single group counterbalanced experimental design experimental design, α = 0.05 and power = 0.80, and an estimated effect size of 0.38 was adopted based on discussions among the authors and the findings of Boukhris et al. [5]. The latter authors reported this value for total distance covered during a 5-m shuttle run test while examining the effects of RF on physical and perceptual outcomes in physically active individuals. Based on these parameters, a minimum of 11 participants was required. Eligibility criteria included a minimum of three years of regular competitive soccer experience; participation in 3 to 4 weekly training sessions (each lasting 60–90 minutes) as confirmed through direct consultation with team coaches and standard weekly schedules; active involvement during the in-season period with at least 80% match participation; a professional contract with a club competing in the 3^rd^ division of the Tunisian national league; non-smoking status and no use of dietary supplements in the past two months (selfreported); no diagnosed sleep disorders (defined as a Pittsburgh sleep quality index or PSQI score < 5); and no current injuries affecting training or performance.

As circadian typology may influence results, participants were assessed using the Horne and Östberg self-assessment questionnaire [31], and only “neither type” chronotypes were included. To control for the performance-enhancing effects of daytime naps during Ramadan [32], participants were instructed to avoid napping. The study was approved by the local ethics committee (CPP SUD:0492/2023) and conducted in accordance with the Declaration of Helsinki. All participants provided written informed consent.

### Methodology

The study was conducted in Sfax, Tunisia, in 2024, during the month of Ramadan, which lasted from March 11 to April 9. Throughout the Ramadan month, the daily fasting from dawn to sunset lasted ~14 hours. Table 1 presents the environmental conditions during the SSG sessions for the study period.

**TABLE 1:** Environmental conditions before and during Ramadan month

Trials	Before Ramadan at 15:00	Before Ramadan at 21:00	During Ramadan at 15:00	During Ramadan at 21:00
Ambient temperature (ºC)	19.0 ± 1.4	16.0 ± 1.4	22.5 ± 0.7	18.5 ± 2.1
Relative humidity (%)	60.0 ± 2.8	70.5 ± 3.5	58.0 ± 2.8	65.0 ± 5.6

Before data collection, players participated in two familiarization sessions to ensure that they gained experience with the experimental procedure. During this period, anthropometric measurements were recorded using a Tanita DC-430MA impedance meter (Tanita Europe BV, UK) and a stadiometer (Tanita Europe BV, UK). Participants’ maximum HR (HRmax) and estimated maximal oxygen uptake (or ${\dot{\text{V}}\text{O}}_{2\max}$) were determined 10 days before the start of Ramadan month, using the Yo-Yo Intermittent Recovery Test Level 1 [33]. Dietary intake was self-reported over three consecutive days, on both occasions, one week before Ramadan month and during the fourth or final week of Ramadan month.

In this within-subjects, counterbalanced study design, each participant completed four SSG sessions scheduled with at least 72 hours of passive recovery period in-between. One week prior to the Ramadan month, two control sessions were conducted at 15:00 and 21:00 in a fed state (i.e., BR15_fed_ and BR21_fed_, respectively). Two experimental sessions (fed and fasted state) were conducted at 15:00 and 21:00 during the last week of Ramadan month (i.e., DR15_fasted_ and DR21_fed_, respectively). The 21:00 sessions were conducted approximately 2 hours and 15 minutes after the day’s fast was broken, i.e., players ingest food and fluids. This evening training time during the Ramadan month is deemed optimal for Ramadan-fasted athletes to maintain adequate hydration and nutrition during training [28, 34]. Total sleep time (TST) measured via actigraphy did not differ significantly between the fed and fasted states, either before Ramadan (474 ± 83 vs. 473 ± 90 minutes; p = 0.141) or during Ramadan (416 ± 96 vs. 378 ± 79 minutes; p = 0.954). Throughout the study, players maintained their usual training loads, with no additional sessions or matches, as confirmed through coordination with each team’s coaching staff. Experimental sessions were scheduled between match-day (MD) MD-4 and MD-3 (i.e. 4 and 3 days before the match) to avoid interference from match-related fatigue. The team’s fitness coach deliberately adjusted the training program to ensure a consistent and low-load environment during the testing period. All sessions were conducted as free play without tactical constraints; the same players participated in all sessions to ensure consistency in team dynamics and minimize variability related to competitiveness or unfamiliarity among teammates. Players’ positions remained the same throughout the SSG training sessions, as positional variation could introduce variability in physical output. No specific tactical instructions were provided during the SSG to replicate realistic competitive conditions and preserve ecological validity.

**FIG. 1:**
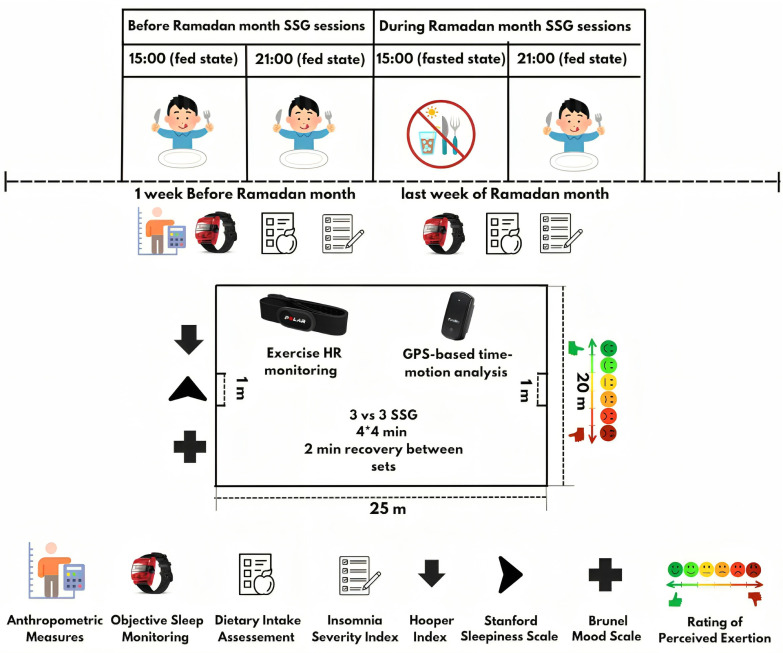
Schematic representation of the study design.

Figure 1 shows the schematic representation of the study design. Time-motion metrics and exercise HR during SSG were assessed using a GPS device and a HR monitor. Ratings of perceived exertion (RPE) [35] were collected ~30 minutes after each SSG session. Sleep was objectively assessed using a wrist-worn actigraphy device for seven consecutive days before the start of the RF month and during the final seven days of Ramadan month. Additionally, players completed the Arabic version of the Insomnia Severity Index (ISI) questionnaire [36] to assess subjective symptoms of insomnia, which are often not fully captured by objective tools, seven days before Ramadan and on the last day of the month. Approximately thirty minutes before the SSG testing sessions (between 14:20–14:45 for the BR15_fed_ and DR15_fasted_ sessions, and between 20:20–20:45 for the BR21_fed_ and DR21_fed_ sessions), participants completed the Stanford Sleepiness Scale (SSS) [37], the Hooper Index questionnaire [38], and the Arabic version of the Brunel Mood Scale (BRUMS) [39]. All questionnaires were administered in paper format under supervision in a quiet, controlled environment. The same researcher conducted and monitored each session to ensure consistency. To avoid any interference of the attending researcher during the survey filling session, all participants received standardized verbal instructions, which were identical across all sessions. Responses were collected using unique participant codes to maintain confidentiality while allowing for longitudinal tracking across all SSG sessions.

### Assessment procedures

#### Nutritional assessments

Participants were instructed to document their dietary intake in detail using a structured food diary. These records were subsequently analysed by a qualified nutritionist using the food composition tables published by the Tunisian National Institute of Statistics (1978) to calculate macronutrient content and total fluid intake [40].

#### Small-sided soccer game (SSG)

The SSG training program consisted of four 4-minute sets of 3 vs. 3 SSG on a 20×25-meter pitch, with a 2-minute passive recovery between sets. Points were scored by scoring small goals (1 m×0.5 m). Each session began with a ~15-minute warm-up (low-intensity running, striding, stretching) and ~5 minutes of ball play. Two coaches positioned around the pitch motivated players and ensured continuous play by providing new balls as needed. At the end of the SSG session, players completed a cool-down and return to a resting phase.

#### Time-motion metrics

Players’ time-motion metrics were monitored via a 10 Hz GPS device (FieldWiz, ASI, Switzerland), which has demonstrated high accuracy in measuring movements and displacements in team sports [42]. The GPS sensors were secured in a vest on each participant’s upper back, with each player consistently using the same unit for enhanced reliability. The devices were activated 15 minutes before the SSG sessions, and the data were analyzed immediately using the manufacturer’s proprietary software package (Fieldwizz, ASI, Lausanne, Switzerland) [42]. Locomotor performance metrics included the total distance covered and distances achieved within four speed zones: walking (0–6.9 km/h), low-intensity running (7.0– 12.9 km/h), moderate-intensity running (13.0–17.9 km/h), and high-intensity running (> 18.0 km/h). The total numbers of accelerations (> 3.0 m/s^2^), decelerations (< −3.0 m/s^2^), and high-intensity running (> 18.0 km/h) were also quantified. These thresholds were consistent with previous SSG research [43]. Periods of inactivity or pauses between sets were excluded from the GPS data analysis.

#### Exercise heart rate responses

During each training session, exercise HR was recorded continuously at 5 Hz using the Polar Team Sports System (Polar Electro Oy, Finland). Relative exercise intensity (as a % of HRmax) was calculated based on each player’s maximum HR using the following formula: exercise HR/ maximum HR *100 [41].

#### Actigraphy registration and analysis

For sleep data, an ActiGraph activity monitor (wGT3X-BT, ActiGraph LLC, USA) was worn on the non-dominant wrist, and the data were analyzed using ActiLife software (version 6.13.4, ActiGraph LLC, FL, USA). Sleep periods, recorded in sleep diaries, were manually entered into ActiLife. The analyzed parameters included sleep onset time, wake time, total sleep time, sleep efficiency, and sleep latency, as detailed in Kerkeni et al. [13].

#### Psychometric and perceptual measures

The ISI questionnaire was used to assess insomnia severity, with the validated Arabic version [36] (Cronbach’s α = 0.86) applied. Scores classified insomnia as ‘no insomnia’ (0–7), ‘subthreshold insomnia’ (8–14), ‘moderate insomnia’ (15–21), or ‘severe insomnia’ (22–28) [44]. The SSS [37] assessed subjective sleepiness on a 7-point scale (1 = highly active; 7 = highly tired). The Hooper wellness Index [38] evaluated fatigue, stress, delayed-onset muscle soreness, and sleep quality, with participants rating each component on a 1–7 scale, where 1 = ‘very good’ (sleep) or ‘very low’ (stress, fatigue, delayed onset muscle soreness (DOMS)) and 7 = ‘very bad’ (sleep) or ‘very high’ (stress, fatigue, DOMS). The RPE was administered with the Borg CR10 scale [14], ranging from 0 (no exertion) to 10 (extremely hard), using the French-validated version [35]. The BRUMS [45] assessed mood states using the validated Arabic version ([39]; with Cronbach’s α > 0.70), which includes six subscales (i.e., fatigue, anger, vigor, confusion, depression, tension). The 24 items were rated on a 5-point Likert scale, with subscale scores ranging from 0 to 16 [45].

### Statistical analyses

All the statistical analyses were performed using STATISTICA (Statistica Kernel version 12; Stat Software; France). The Shapiro–Wilk test was employed to assess the normality of the data. Non-normally distributed parameters with repeated measures (high-intensity running distance, number of acceleration, number of high intensity running, stress, sleep duration, sleep efficiency, and sleep latency) were analyzed using the Friedman non-parametric test. When appropriate, pairwise comparisons were conducted using the Wilcoxon test. Normally distributed data (energy intake, protein, fat, carbohydrates, sleep onset time, wake time, and ISI score) were analyzed using parametric tests. Student’s t-test was employed to assess variations in these parameters between two experimental periods. However, a Wilcoxon test was used to compare total sleep time, sleep efficiency, and sleep latency scores between the experimental and control periods.

Two-way ANOVA was conducted on normally distributed data with repeated measures (absolute and relative exercise HR, total distance, walking distance, low-intensity distance, moderate intensity distance, decelerations, overall Hooper index and its components, RPE, sleepiness, and BRUMS components) to assess the effects of condition (fed vs. fasted), period (pre-Ramadan vs. Ramadan), and their interaction. When applicable, post-hoc comparisons were conducted using Bonferroni correction. Cohen’s d and its corresponding 95% confidence interval (CI) was calculated to determine effect sizes for paired comparisons, with the following interpretation: 0.2 ≤ 0.5 as ‘small’, 0.5 ≤ 0.8 as ‘moderate’, and ≥ 0.8 as ‘large’ [46]. For the normally distributed parameters with repeated measures, effect sizes were determined using partial eta-squared (ƞp^2^). Partial eta-squared values were interpreted as follows: < 0.01 for ‘small’, 0.01 to < 0.06 for ‘moderate’, and 0.06 to 0.14 for ‘large’ [47]. Statistical significance was set at p < 0.05 for all analyses.

## RESULTS

### GPS-derived time-motion metrics

Table 2 shows changes in time-motion metrics and exercise HR across fed and fasted states during Ramadan month.

**TABLE 2:** Mean exercise heart rate (HR) values, percentage of maximum heart rate (%HR_max_), and GPS-based time-motion metrics recorded Before and During Ramadan under fed and fasted conditions.

Indicator	Before Ramadan 15:00 (fed)	During Ramadan 15:00 (fasted)	Before Ramadan 21:00 (fed)	During Ramadan 21:00 (fed)	Condition			Period			Condition × Period		
					F	P-value	ƞp^2^	F	P-value	ƞp^2^	F	P-value	ƞp^2^
HR (b/min)	176.5 ± 4.1	168.2 ± 3.7^*^	172.8 ± 4.3^*^	171.4 ± 4.6	34.98	<0.001	0.76	0.03	0.85	0.003	23.61	<0.001	0.68
%HR_max_	89.1 ± 2.0	84.9 ± 1.9^*^	87.2 ± 2.3^*^	86.5 ± 2.4	34.91	<0.001	0.76	0.03	0.86	0.002	23.59	<0.001	0.68
Total distance (m)	1422 ± 144	1363 ± 200	1448 ± 208	1457 ± 187	0.38	0.547	0.03	6.06	0.031	0.35	0.93	0.353	0.07
Walking during the 4 × 4 min (m)	739 ± 87	739 ± 67	738 ± 51	734 ± 40	0.01	0.910	0.001	0.06	0.804	0.005	0.01	0.895	0.001
Low intensity running during the 4 × 4 min (m)	518 ± 124	442 ± 155	507 ± 165	498 ± 124	1.28	0.281	0.10	0.81	0.387	0.06	1.44	0.254	0.11
Moderate intensity running during the 4 × 4 min (m)	143 ± 56	156 ± 61	164 ± 52	182 ± 63	1.35	0.269	0.10	9.35	0.010	0.45	0.02	0.877	0.002
High intensity running during the 4 × 4 min (m)	27 ± 24	25 ± 23	33 ± 29	38 ± 24	Statistics for parameters with non-normal distribution are presented in the text below.								
Number of accelerations	11.0 ± 5.6	9.7 ± 4.6	9.7 ± 4.6	11.4 ± 2.6									
Number of decelerations	6.9 ± 1.4	7.4 ± 3.7	6.8 ± 4.1	11.3 ± 3.8^#&^	11.45	0.006	0.51	3.24	0.099	0.22	12.27	0.004	0.52
Number of high intensity runs (> 18 km/h)	3.4 ± 2.6	3.7 ± 2.6	5.8 ± 3.7	5.7 ± 3.6	Statistics for parameters with non-normal distribution are presented in the text below								

* Significant difference compared to before Ramadan at 15:00;

# significant difference compared to during Ramadan at 15:00;

& significant difference compared to before Ramadan at 21:00; ƞp^2^: partial eta-squared; F = F-statistic from analysis of variance (ANOVA)

The post-hoc Bonferroni analysis revealed that in BR15_fed_, the absolute and relative exercise HR were significantly higher than those recorded in BR21_fed_ (both p = 0.02). Additionally, a significant reduction in the mean absolute and relative exercise HR was observed in DR21_fasted_ compared to BR15_fed_ (p < 0.001 for both). The number of decelerations in DR21_fasted_ was significantly higher compared to both BR21_fed_ (p < 0.001) and DR15_fed_ (p = 0.003). For total distance, and distance covered at moderate-intensity running, the post-hoc correction analysis revealed no significant differences between before Ramadan and during the Ramadan month or between the fed and fasted conditions. There were also no significant effects on distance covered at high-intensity running (test = 3.08; p = 0.37; Kendall’s W = 0.08), the number of high intensity running (test = 6.40; p = 0.09; Kendall’s W = 0.17), or the number of accelerations (test = 1.61; p = 0.65; Kendall’s W = 0.04).

### The Hooper index

Table 3 shows the Hooper index parameters measured before and during Ramadan month.

**TABLE 3:** Hooper index parameters (i.e., fatigue, stress, sleep, muscle soreness, and overall Hooper index), measured before and during Ramadan under fed and fasted conditions.

Indicator	Before Ramadan 15:00 (fed)	During Ramadan 15:00 (fasted)	Before Ramadan 21:00 (fed)	During Ramadan 21:00 (fed)	Condition			Period			Condition × Period		
					F	P-value	ƞp^2^	F	P-value	ƞp^2^	F	P-value	ƞp^2^
Sleep (a.u)	2.8 ± 1.1	3.8 ± 1.1	3.8 ± 0.9	3.6 ± 1.0	3.33	0.094	0.23	4.06	0.068	0.27	6.76	0.024	0.38
Stress (a.u)	2.5 ± 0.8	3.7 ± 1.2^*^	3.1 ± 0.9	3.4 ± 1.2
Fatigue (a.u)	3.3 ± 1.1	3.6 ± 0.8	3.3 ± 1.1	3.6 ± 1.2	0.88	0.367	0.07	1.01	0.899	0.001	0.04	0.845	0.003
Muscle soreness (a.u)	2.8 ± 1.1	3.8 ± 1.1^*^	3.2 ± 0.9	3.5 ± 1.2	3.66	0.081	0.25	0.02	0.880	0.002	6.49	0.027	0.371
Overall Hooper index (a.u)	2.9 ± 0.5	3.7 ± 0.5^*^	3.3 ± 0.3	3.5 ± 0.4	13.85	0.003	0.55	1.43	0.256	0.11	22.63	<0.001	0.67

ƞp^2^: partial eta-squared; F = F-statistic from ANOVA

* Significant difference compared to before Ramadan at 15:00

^#^Significant difference compared to during Ramadan at 15:00

^&^Significant difference compared to before Ramadan at 21:00

Post-hoc Bonferroni analysis revealed muscle soreness scores were significantly higher in DR15_fed_ compared to BR15_fed_ (*p* < 0.001). Additionally, there were no significant differences in the sleep component of the Hooper Index between the before and during Ramadan month, or between the fed and fasted conditions.

For the players’ wellness, post-hoc analysis indicated that the overall Hooper index was significantly higher (*p* < 0.001) for DR15_fed_ compared to BR15_fed_.

Friedman’s test revealed a significant effect on stress levels. Wilcoxon pairwise comparisons further indicated that stress levels were significantly higher in DR15_fed_ compared to BR15_fed_ (*p* = 0.007).

### Objective sleep metrics

Objective sleep metrics recorded before and during Ramadan month are presented in Table 4.

**TABLE 4:** Objective sleep metrics recorded before and during Ramadan under fed and fasted conditions.

Actigraphy variables	Before Ramadan	During Ramadan	P-value	Test	Effect size	95% CI
Sleep duration (min) Median [IQR]	454 ± 33 451 [428–484]	400 ± 57^*^ 406 [396–444]	0.015	-2.432	0.70	[0.27, 0.88]
Sleep latency (min) Median [IQR]	18.3 ± 6.2 18 [15–19]	19.3 ± 3.3 18 [17–22]	0.372	-0.894	0.25	[-0.73, 0.46]
Sleep efficiency (%) Median [IQR]	95.9 ± 1.4 96.4 [95.4–96.8]	95.0 ± 1.3 95.4 [94.0–96.1]	0.084	-1.729	0.49	[-0.01, 0.83]
Sleep onset time (hh:mm)	00:44 ± 00:38	01:31 ± 00:54	0.071	-1.962	0.56	[-1.17, 0.05]
Wake time (hh:mm) Median [IQR]	08:19 ± 00:23 08:19 [08:23–08:33]	08:01 ± 00:26 08:00 [07:48–08:15]	1.167	1.501	-0.43	[-0.99, 0.13]

* Significant difference compared to before Ramadan

CI: confidence interval; IQR: Interquartile range

Sleep duration was significantly reduced during Ramadan compared to before Ramadan month (*p* = 0.01; Test = -2.432; Cohen’s *d* = 1.16). However, no significant effect was observed on the remaining actigraphic parameters.

### Insomnia levels

The total ISI score significantly increased from before (10.0 ± 2.2 a.u.) to during Ramadan month (11.6 ± 2.4 a.u.) (*p* = 0.003, Cohen’s *d* = 0.69).

### Mood states

Results of the BRUMS recorded before and during Ramadan month are presented in Table 5.

**TABLE 5:** Means, standard deviations, and ANOVA results for Brunel Mood Scale scores before and during Ramadan under fed and fasted states.

	Before Ramadan 15:00 (fed)	Before Ramadan 21:00 (fed)	During Ramadan 15:00 (fasted)	During Ramadan 21:00 (fed)	Condition			Period			Condition × Period		
					F	P-value	ƞp^2^	F	P-value	ƞp^2^	F	P-value	ƞp^2^
Fatigue (a.u)	6.6 ± 1.2	6.7 ± 1.4	7.0 ± 0.9	6.1 ± 1.3	0.23	0.635	0.02	1.31	0.275	0.10	0.74	0.405	0.06
Tension (a.u)	5.1 ± 1.1	5.5 ± 0.9	6.3 ± 1.5	6.0 ± 0.8	4.99	0.047	0.31	0.005	0.946	0.000	1.28	0.280	0.10
Depression (a.u)	5.3 ± 1.2	4.3 ± 1.1	4.6 ± 1.3	4.8 ± 1.1	0.14	0.715	0.01	0.92	0.358	0.07	3.67	0.081	0.25
Confusion (a.u)	3.9 ± 1.3	4.2 ± 1.6	3.5 ± 1.3	4.3 ± 1.9	0.09	0.761	0.008	2.58	0.136	0.19	0.30	0.591	0.02
Vigor (a.u)	6.5 ± 0.9	7.0 ± 1.6	5.8 ± 1.6	7.2 ± 1.0	0.21	0.651	0.01	7.09	0.022	0.39	1.40	0.260	0.11
Anger (a.u)	4.6 ± 1.5	4.8 ± 1.4	5.2 ± 1.6	4.1 ± 1.4	0.00	0.948	0.000	1.48	0.249	0.11	2.27	0.159	0.17

ƞp^2^: partial eta-squared; F = F-statistic from ANOVA

Pairwise comparisons revealed no significant differences between the before and during Ramadan periods or between the fed and fasted conditions for all BRUMS components.

### Dietary intake

Results of the estimated daily dietary intake recorded before and during Ramadan month are presented in Table 6.

**TABLE 6:** Estimated daily dietary intake recorded before and during Ramadan.

	Before Ramadan	During Ramadan	P-value	Test	Effect size	95% CI
Total energy intake (Kcal/day)	2930 ± 314	2799 ± 198	0.332	1.014	0.29	[-0.29, 0.86]
Carbohydrates (g) (%)	325 ± 41 52.1 ± 4.2	298 ± 19 44.7 ± 2.9^*^	0.105 0.002	1.768 3.985	0.51 1.15	[-0.10, 1.10] [-0.39, 1.87]
Protein intake (g) (%)	111 ± 15 17.8 ± 2.3	125 ± 5^*^ 17.9 ± 0.7	0.005 1.000	-3.540 0.000	-1.02 0.00	[-1.71, -0.30] [-0.56, 0.56]
Total fat intake (g) (%)	83 ± 11 29.9 ± 3.6	116 ± 5^*^ 37.4 ± 2.7^*^	<0.001 0.001	-11.776 -4.820	-3.40 -1.39	[-4.89, -1.88] [-2.18, -0.57]
Total water intake (L)	3.8 ± 0.4	3.5 ± 0.3	0.17	1.482	0.42	[-0.17, 1.01]

CI: confidence interval

* Significant difference compared to before Ramadan

There was no significant effect of RF on total energy intake (*p* = 0.33), total water intake (*p* = 0.17), or carbohydrates intake (*p* = 0.10). However, total fat intake (*p* < 0.001) and protein intake (*p* = 0.005) increased during RF.

### Rating of Perceived Exertion (RPE)

**FIG. 2:**
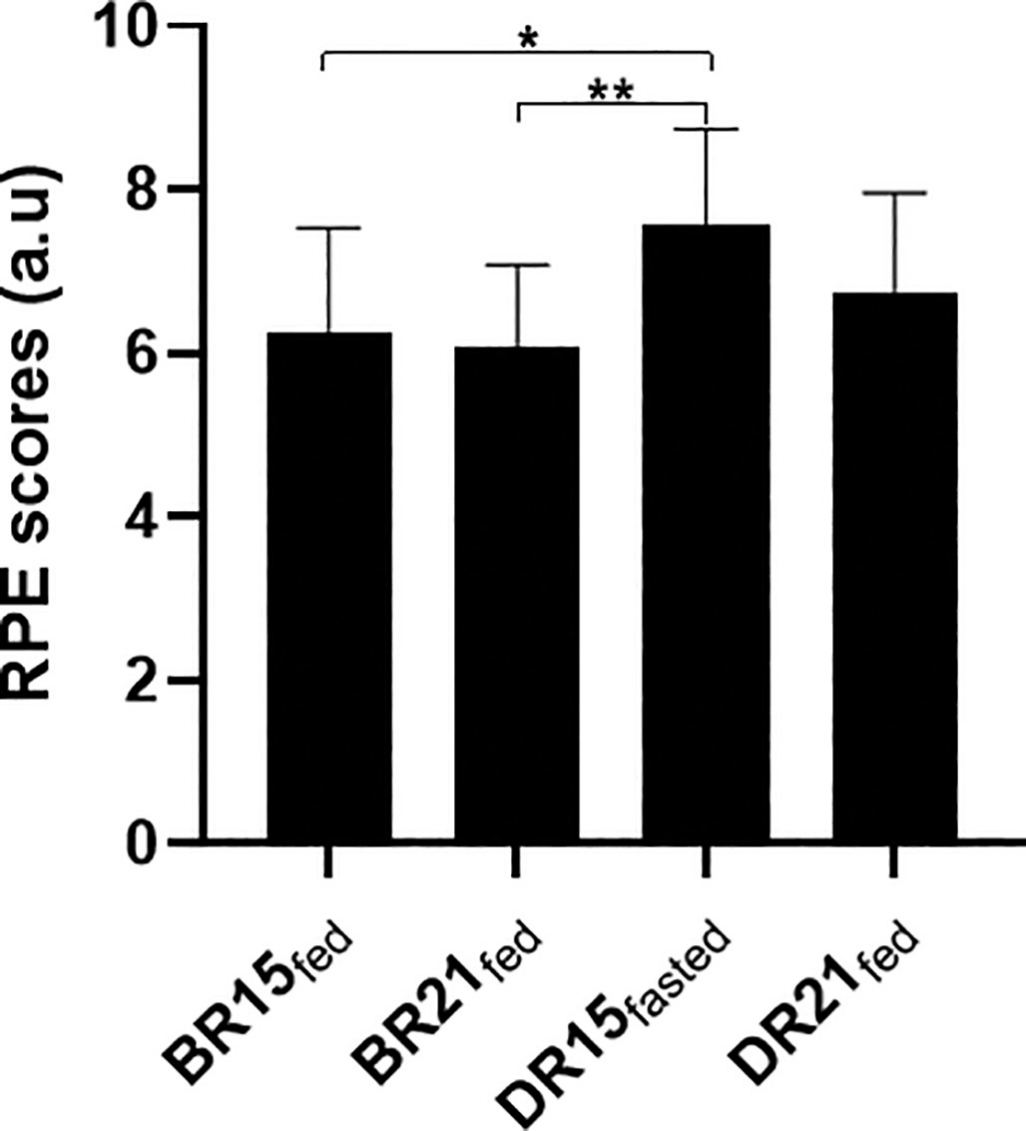
Mean values (± SD) for ratings of perceived exertion (or RPE) values after small-sided games (SSG) in BR15_fed_, BR21_fed_, DR15_fasted_, and DR21_fed_. *p < 0.05; **p < 0.01.

Mean values for RPE are presented in Figure 2. Statistical analyses revealed a significant main effect of condition (*p* = 0.007; F = 10.56; ƞp^2^ = 0.48) and period (*p* = 0.032; F = 6.0; ƞp^2^ = 0.35) on RPE values. The pairwise comparison revealed that RPE values were higher in DR15_fasted_ compared to BR15_fed_ (*p* = 0.011) and BR21_fed_ (*p* = 0.004).

### Pre-SSG daytime sleepiness

**FIG. 3:**
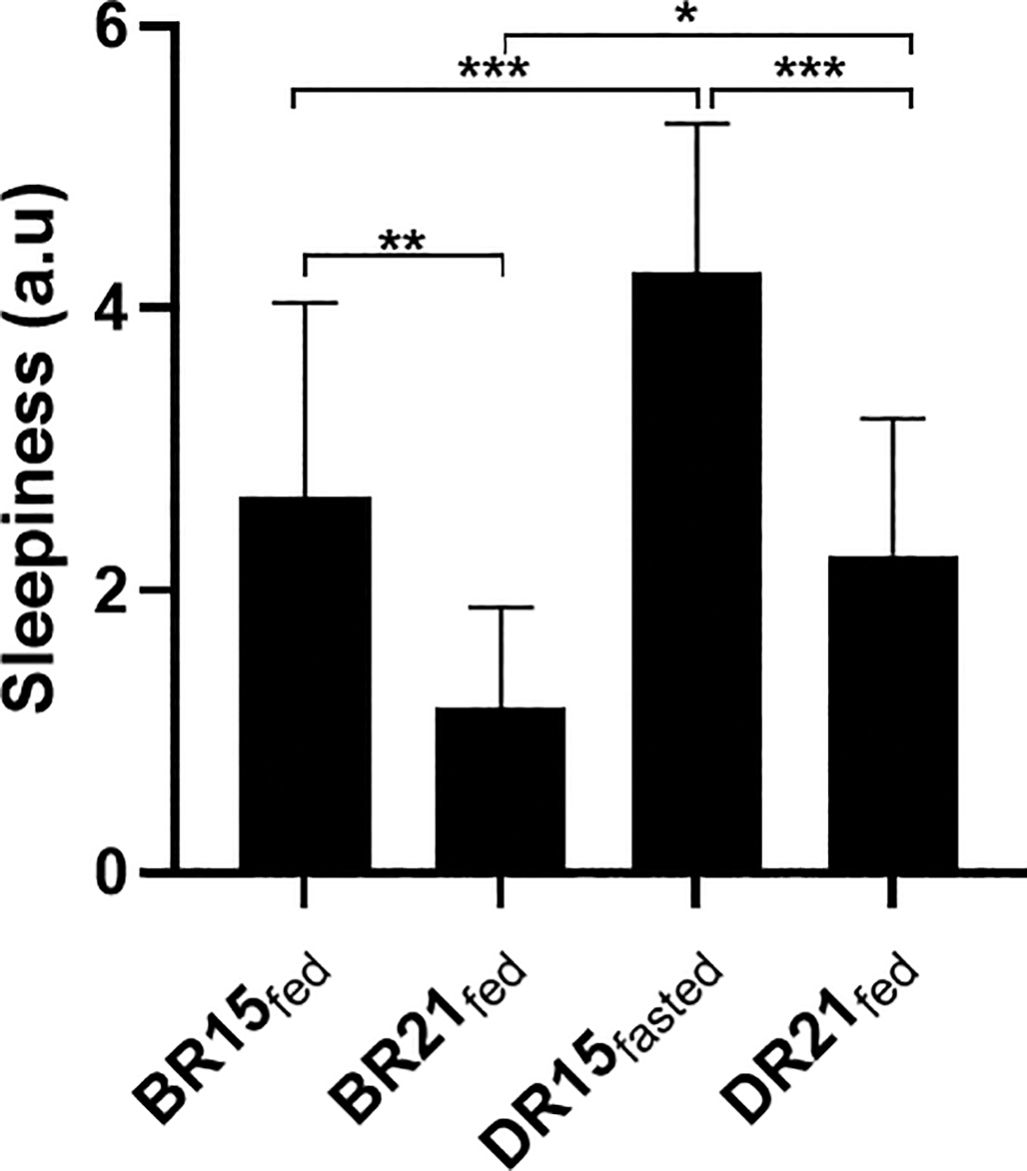
Mean values (± SD) for pre-SSG sleepiness values in BR15_fed_, BR21_fed_, DR15_fasted_, and DR21_fed_. *p < 0.05; **p < 0.01; ***p < 0.001.

The SSS mean values are presented in Figure 3. There was a significant main effect of condition (p = 0.000; F = 20.11; ƞp^2^ = 0.64) and period (p = 0.000; F = 30.50; ƞp^2^ = 0.73) on sleepiness scores. The pairwise comparison indicated that sleepiness scores were higher in BR15_fed_ compared to BR21_fed_ (*p* = 0.001), and higher DR15_fasted_ in comparison with BR15_fed_ (*p* < 0.001). Additionally, sleepiness levels were lower DR21_fed_ compared to DR15_fasted_ (*p* < 0.001), and BR21_fed_ compared to DR21_fed_ (*p* = 0.016).

## DISCUSSION

In this within-subjects counterbalanced study study in semi-professional male soccer players, RF had no significant impact on most GPS parameters during SSG. In contrast, there was an increased number of decelerations during Ramadan in the fed state. Moreover, mean absolute and relative exercise HR decreased during Ramadan in the fed state compared to before Ramadan in the same feeding state. Participants’ RPE, insomnia, and daytime sleepiness scores were higher during RF, suggesting potential alterations in perceptual responses and sleep patterns, with an objectively assessed decrease in sleep duration during Ramadan month. The overall Hooper index score was higher in both the fasted and fed states during RF as compared to baseline levels, indicating an increased psychophysiological strain. RF did not significantly affect BRUMS mood scale components under either fed or fasted conditions, indicating stable mood states throughout the study.

Contrary to our hypothesis, the GPS time-motion metrics remained largely unchanged, except for an increase in the number of decelerations during the fed condition during RF, i.e., DR21_fed_. This contrasts with the previous findings of Aziz et al. [19], who reported significant reductions in total distance in their players’ moderate- and high-velocity running during a 90-minute Ramadan-fasted soccer match. These discrepancies may be attributed to several key methodological differences. First, the significantly shorter exercise duration in our study (i.e., 16 minutes of SSG) compared to the ~90-minute duration of a full match likely resulted in substantially lower cumulative fatigue. Second, differences in the competitive level and training status of the participants may have influenced the divergent findings; Aziz’s participants were sub-elite players [19], whereas those in the present study were semi-professionals.

Additionally, our data showed no changes in total energy intake throughout Ramadan, a finding consistent with the study of Bouzid et al. [48] in soccer players. Although the difference was not statistically significant, the small effect size and the wide CI suggest uncertainty about the direction and magnitude of the effect. This relative stability in energy intake may have partially contributed to the maintenance of SSG time-motion metrics. Collectively, these results suggest that implementing appropriate hydration and nutritional strategies outside of fasting hours can mitigate the potential negative effects of RF on athletes’ short-duration GPS-based time-motion metrics [34]. It is also worth noting that the degree or level of the athletes’ religious beliefs may inspire resilience and motivation, potentially mitigating any expected decline in performance [49]. Interestingly, the increased number of decelerations in DR21_fed_ may have practical relevance. Decelerations are as frequent as accelerations in soccer players [50] and contribute significantly to neuromuscular and mechanical load given their high eccentric demands [50, 51]. This increase may reflect greater player engagement in the fed evening condition, potentially influenced by improved hydration, energy availability, and/or alertness post-Iftar, i.e., breaking of the day’s fast. It appears that training sessions scheduled after Iftar during Ramadan may impose higher neuromuscular loads, particularly through repeated decelerations, even in the absence of changes in the other time-motion metrics.

A significant decrease was observed in the mean absolute and relative exercise HR during Ramadan at 15:00 (i.e., DR15_fasted_) compared with the same time of day before Ramadan (i.e., BR15_fed_). This decrease may be explained by fasting-induced inhibition of catecholamine secretion and reduced venous return, both of which contribute to lower sympathetic activity [52]. Indeed, these physiological responses can reduce blood pressure, HR, and cardiac output [52]. Zoladz et al. [53] demonstrated that in the fasted state, elevated plasma norepinephrine increases systemic vascular resistance, activating arterial baroreceptors and vagal tone, potentially contributing to the HR reduction we observed. Despite a lower exercise HR, athletes maintained their time-motion activity in the Ramadan-fasted condition, probably through an effective pacing strategy [34]. For the evening SSG session, i.e., DR21_fed_, however, nutrition and hydration are consequently restored via the breaking of the day’s fast, i.e., Iftar meal, possibly contributing to similar cardiovascular responses as the before Ramadan condition, i.e., BR21_fed_. Our findings are consistent with those of previous studies; for example, Brini et al. [7] reported decreased exercise HR during small-sided basketball games conducted during Ramadan observance. Similarly, another group of investigators [54] reported a similar decline in exercise HR in response to submaximal exercise during Ramadan in sedentary males. However, contrasting results were reported by another Ramadan study of Aziz et al. [55], who found higher exercise HR values during submaximal-intensity running at 65% ${\dot{\text{V}}\text{O}}_{2\mathrm{peak}}$ in a fasted state, in a laboratory setting of 20–24 ºC ambient temperature and 55–60% relative humidity conditions, among physically active men. The authors attributed this increase to hypohydration at the start of exercise rather than hypohydration occurring during the 30 minutes exercise itself [55]. In contrast, significant hypohydration was unlikely in the present study, given the similarly short duration of the SSG sessions as well as the less stressful environmental conditions in the present study (see Table 1), which would not be expected to induce substantial fluid loss [56]. Moreover, dietary records (see Table 6) indicated that total water intake remained consistent and similar between before Ramadan and during Ramadan month, further minimizing the likelihood that hydration status would have had influenced our observed cardiovascular responses.

Similar to exercise HR, the post-session RPE is widely used to assess exercise intensity [55]. In our study, RPE scores recorded in the fasted state during Ramadan were higher than those of before Ramadan, aligning with previous findings reporting elevated RPE during exercise in the Ramadan-fasted state when compared to control periods across various sporting contexts, including simulated soccer matches [19], small-sided basketball training programs [7], and maximal exertion field tests [16]. In contrast, RPE remained constant in all other fed conditions, either before and during Ramadan observance, likely because nutrition and hydration levels in these conditions are restored, ensuring similar physiological and perceptual responses to exercise.

The increased post-session RPE suggests that fasted-state exercise was perceived as more physically and mentally demanding, likely due to increased fatigue and sleep disturbances associated with the observance of RF. Supporting this view, Kerkeni et al. [13] observed higher levels of insomnia symptoms and daytime sleepiness among student-athletes during RF, which were linked to a mean reduction of ~45 minutes in total sleep duration; and their mean total sleep of 6 h 40 minutes was also well below the 9–10 hours recommended for athletes [57]. In our study, the reduction in sleep duration during RF was both statistically significant and accompanied by a moderate effect size, indicating a meaningful change. The 95% CI suggests that the true effect may range from small to large, reflecting limited precision but reinforcing the potential relevance of sleep reduction in the context of athlete readiness and recovery. Alternatively, the increased RPE observed during RF may be attributed in part to a placebo (or more aptly, nocebo) effect, as the fasted condition could not be concealed from participants [55]. Nonetheless, GPS-based time-motion activity remained unaffected, possibly due to preserved sleep efficiency and partial adaptation to fasting. It could also be argued that the extent of sleep loss—equivalent to half a sleep cycle—was unlikely to be sufficient to impair short-duration SSG time-motion activity.

Although RPE scores were elevated during Ramadan at 15:00 compared to before Ramadan at the same time of day, exercise HR was significantly reduced, indicating a mismatch between perceived effort and physiological response. This discrepancy may be explained by increased psychological stress associated with RF, as indicated by both higher stress levels measured via the Hooper Index questionnaire. Overall, the results of the present study appear to indicate that neither HR nor RPE alone may reliably capture actual exercise intensity in the fasted state. Consequently, GPS-derived data, as a direct measure of external load, should rather be considered when monitoring exercise effort during Ramadan [55].

Interestingly, the participants’ mood states, as measured by the BRUMS, remained relatively stable during the Ramadan month; this contrasts with the findings of the systematic review and meta-analysis conducted by Trabelsi et al. [2] who reported increased fatigue and reduced vigor during RF in athletes. The lack of mood disturbances observed in our study, despite the physiological and perceptual challenges of observing RF, may result from both psychological and physiological adaptations. Athletes regularly exposed to repeated fasting are likely to develop coping strategies and resilience that help regulate their emotions [55]. Given the mediating role of mood in athletic performance [56], its preservation may reflect athletes’ motivation to maintain their exercise performance during a religiously meaningful period. Hence within this context, the inherently sociocultural support that Ramadan-fasted athletes experience may buffer negative emotions and at the same time reinforce the self-discipline and purposefulness nature of observing RF [58].Thus, mood stability during Ramadan month likely reflects successful adaptation rather than absence of stressors.

Another important factor to consider when comparing daytime and evening training sessions in the present study is the prevailing environmental conditions during the SSG sessions. Since the sessions were conducted outdoors, the cooler ambient temperatures in the evening may have helped mitigate athletes’ physiological responses, contributing to lower psychophysiological strain in the DR21_fed_ relative to the DR15_fasted_ sessions.

### Practical applications

Short-duration SSG can be maintained whether performed in a fed or fasted state during RF without significant alterations in GPSderived time-motion metrics. However, coaches should carefully consider the physiological and perceptual differences when planning training in the Ramadan month. In particular, training intensity and volume should be moderated during fasted-state sessions [59]. These sessions may be better suited for technical or tactical drills rather than high-intensity conditioning. In contrast, physically demanding or high-intensity sessions should be scheduled post-Iftar, when fasting athletes are rehydrated and refueled, to reduce the risk of accelerated fatigue and maladaptation. Additionally, training periodization during the Ramadan month may require a strategic redistribution of weekly training loads, with lower-load sessions placed earlier in the day, and higher-load sessions timed post-Iftar period [59]. Additionally, internal load should be monitored consistently using simple and non-invasive tools such as RPE scales, wellness questionnaires, and sleep tracking. These measures can help detect early signs of physical and possibly mental fatigue and maladaptation, enabling timely adjustments to training loads during the Ramadan period. Moreover, since cardiovascular and perceptual responses may not reliably reflect time-motion metrics under fasting conditions, coaches are advised to prioritize external load metrics (i.e., GPS-derived data) when prescribing training and designing recovery protocols.

To support recovery and mitigate acute strain, individualized strategies should be implemented [27]. These include ensuring adequate post-Iftar hydration and nutritional intake, promoting good sleep hygiene, and incorporating both passive (e.g., rest, relaxation) and active (e.g., stretching) recovery methods [27].

### Limitations

One of the main limitations of the present study was the absence of a control group, a common challenge in RF-based studies [12]. Obviously, it was not ethically feasible to request Muslim athletes to abstain from fasting during Ramadan month; and despite our efforts, we did not find enough non-fasting players within the same squad to constitute a control group. Another limitation was the lack of hydration status assessment, which could have provided relevant information on the potential impact of fluid balance on exercise performance and subjective effort during RF. Although the percentage of heart rate reserve (%HRR) is considered a more a more reliable indicator of HR [60, 61], %HRmax was used in this study given its widespread use in SSG research and practical advantages in field-based protocols [24, 43, 62, 63]. Future studies should consider including %HRR alongside %HRmax to improve physiological precision and sensitivity. In our current study, Iftar meals were not standardized across participants to respect individual dietary habits and cultural traditions; this may have introduced some variability in postprandial responses but contributed to enhanced ecological validity of the study. Additionally, although dietary records indicated no significant difference in total daily water intake between periods, we cannot conclude with certainty that hydration status remained stable during Ramadan month. This uncertainty highlights the need for future studies to assess hydration using physiological biomarkers such as urine osmolality, urine specific gravity, or plasma osmolality [64]. Finally, our study focused on semi-professional male soccer players and included measurements only before and during the last week of Ramadan, which may limit the generalizability of the findings to professional or female players and may not capture variations occurring throughout the month.

## CONCLUSIONS

Despite reductions in sleep duration and alterations in perceptual responses, GPS-based time-motion metrics remained largely unaffected by the observance of RF, indicating that short-duration physical performance in small-sided games can be maintained in both fed and fasted states.

## Data Availability

The data supporting the findings of this study are available from the corresponding author upon reasonable request.
